# Inhibition of matrix metalloproteinases attenuates brain damage in experimental meningococcal meningitis

**DOI:** 10.1186/s12879-014-0726-6

**Published:** 2014-12-31

**Authors:** Susanna Ricci, Denis Grandgirard, Michael Wenzel, Tiziana Braccini, Paola Salvatore, Marco R Oggioni, Stephen L Leib, Uwe Koedel

**Affiliations:** Department of Medical Biotechnologies, Laboratory of Molecular Microbiology and Biotechnology (LA.M.M.B.), Ospedale Santa Maria alle Scotte (V lotto, piano 1), University of Siena, Viale Bracci, Siena 53100 Italy; Institute for Infectious Diseases, University of Bern, Bern, 3010 Switzerland; Department of Neurology, Klinikum Großhadern, Ludwig-Maximilians University, Munich, 81377 Germany; Department of Molecular Medicine and Medical Biotechnology, Federico II University Medical School, Naples, Italy; Department of Genetics, University of Leicester, Leicester, LE1 7RH UK; The ESCMID Study Group for Infectious Diseases of the Brain (ESGIB), Basel, Switzerland; Columbia University, Biological Sciences, 901 NWC Building, 550 West 120th Street, New York, 10027 NY USA

**Keywords:** Neisseria meningitidis, Meningococcal meningitis, Mouse model, Brain damage, Matrix metalloproteinases, Metalloproteinase inhibitors

## Abstract

**Background:**

Approximately 7% of survivors from meningococcal meningitis (MM) suffer from neurological sequelae due to brain damage in the course of meningitis. The present study focuses on the role of matrix metalloproteinases (MMPs) in a novel mouse model of MM-induced brain damage.

**Methods:**

The model is based on intracisternal infection of BALB/c mice with a serogroup C *Neisseria meningitidis* strain. Mice were infected with meningococci and randomised for treatment with the MMP inhibitor batimastat (BB-94) or vehicle. Animal survival, brain injury and host-response biomarkers were assessed 48 h after meningococcal challenge.

**Results:**

Mice that received BB-94 presented significantly diminished MMP-9 levels (*p* < 0.01), intracerebral bleeding (*p* < 0.01), and blood-brain barrier (BBB) breakdown (*p* < 0.05) in comparison with untreated animals. In mice suffering from MM, the amount of MMP-9 measured by zymography significantly correlated with both intracerebral haemorrhage (*p* < 0.01) and BBB disruption (*p* < 0.05).

**Conclusions:**

MMPs significantly contribute to brain damage associated with experimental MM. Inhibition of MMPs reduces intracranial complications in mice suffering from MM, representing a potential adjuvant strategy in MM post-infection sequelae.

**Electronic supplementary material:**

The online version of this article (doi:10.1186/s12879-014-0726-6) contains supplementary material, which is available to authorized users.

## Background

*Neisseria meningitidis* is an obligate human pathogen that colonises the nasopharyngeal mucosa of 10-30% individuals, with carriage rates increasing with age from small children to young adults. In a minor proportion of carriers, asymptomatic colonisation may progress into invasive meningococcal disease. The clinical manifestations of meningococcal disease (MD) vary considerably, ranging from transient bacteremia to meningitis and/or fulminant sepsis [1],[2]. The most common clinical presentation of MD is meningococcal meningitis (MM), which can affect 30-60% of infected patients, with adolescents and young adults being the age group at higher risk [3],[4]. MM is associated with 5-16% mortality [3]. Approximately 7% of MM survivors suffer from long-term neurological sequelae (*i.e.*, hearing loss, cognitive impairment, motor deficits, seizures) due to brain damage in the course of meningitis [5].

Both the host immune response to the pathogen and the direct cytotoxicity of bacterial components are responsible for brain tissue damage [6],[7]. Polymorphonuclear cells (PMNs), macrophages and microglia that are recruited to the damaged tissue release reactive oxygen species, matrix metalloproteinases (MMPs), cytokines and excitatory amino acids, finally leading to energy failure and cell death [6],[7].

MMPs are Zn^2+^-dependent peptidases playing a major role in inflammation, innate immunity and tissue turnover. Not only do they target extracellular matrix proteins, but act as sheddases on non-matrix proteins, such as cyto- and chemokines (and their receptors) and antimicrobial peptides [8]. A pathogenic role for MMPs has been suggested for various diseases, including arthritis, cancer, multiple sclerosis, stroke and bacterial meningitis (BM) [9],[10]. MMP levels are increased in the cerebrospinal fluid (CSF) of both adults and children with BM [11]-[13], and high concentrations of the gelatinase MMP-9 are considered a risk factor for the development of neurological sequelae [12]. Several experimental models of pneumococcal meningitis (PM) have demonstrated that MMPs contribute to brain damage by participating in blood-brain barrier (BBB) breakdown, PMN recruitment, shedding of cytokines as well as their receptors, and cortical damage [14]-[16]. Therapy with MMP inhibitors in experimental PM significantly improved animal mortality, neuronal injury, hearing loss, seizures and learning abilities [17]-[21]. In contrast to plentiful data on MMPs in PM models, the importance of MMPs in experimental MM has hardly been reported [11],[22], due to the recognised difficulties of mimicking MD/MM in animal models.

Humans are the only natural hosts for *N. meningitidis* due to the species-specificity of iron meningococcal uptake systems and surface structures. Current animal models of MD consist in mice or rats infected either via the intraperitoneal or intranasal route. In order to compel meningococcal replication in the rodent host, several strategies were attempted, including the use of high bacterial inocula, neonatal or immunocompromised animals, animal passage prior to infection, or administration of an exogenous source of iron [23]-[27]. Transgenic mice for human CD46 [28] or transferrin [29] have also been employed to overcome shortcomings of traditional systems. Recently, an MM mouse model based on intracisternal infection of adult mice with group C *N. meningitidis* has been developed [30]. Meningococci efficiently multiplied in the brain and induced meningitis with features mimicking those present in humans [30].

In this study, we have developed a novel mouse model of MM-induced brain damage and demonstrated the therapeutic efficacy of batimastat, a broad MMP inhibitor, in experimental MM. Evaluation of cerebral haemorrhage, BBB breakdown and apoptosis in the hippocampus showed reduced intracranial complications in mice treated with batimastat, suggesting a key role for MMPs in brain damage associated with MM.

## Methods

### N. meningitidis

The serogroup C 93/4286 isolate, belonging to the ET-37 hypervirulent lineage, was cultured on chocolate GC agar media (Oxoid, Milano, Italy) or CG broth (Oxoid) supplemented with 1% (v/v) Vitox (Oxoid) at 37°C in 5% CO_2_. Inocula for mouse challenge were prepared by cultivating bacteria in GC broth until mid-logarithmic phase. Viable counts were performed, and bacteria were frozen at -80°C with 10% glycerol until use.

### Mice

Six-weeks-old female BALB/c mice were obtained from Charles River (Calco, Italy). Animal experiments were authorised by the local ethics committee (Comitato Etico Locale, Azienda Ospedaliera Universitaria Senese, 21.05.2012) and the Italian Ministry of Health (document no. 131/2013, 30.05.2013), and were carried out according to institutional guidelines.

### Model of MM-induced brain damage

The model of brain damage was developed based on a previously reported MM model in the mouse [30]. Bacteria were thawed, centrifuged for 5 min at 1800 x *g*, and suspended in GC broth with iron dextran (5 mg/kg; Sigma-Aldrich, Milano, Italy) at a final concentration of 10^8^ cfu/ml. Approximately 2 h prior to infection, animals were injected intraperitoneally (i.p.) with iron dextran (250 mg/kg). Mice were lightly anaesthetised by i.p. injection with Zoletil 20 [(tiletamine and zolazepam hydrochloride), 15 mg/kg; Virbac Srl, Milano, Italy] and Xilor [(xylazine 2%), 4 mg/kg; Bio 98 Srl, Milano, Italy] and infected by the intracisternal (i.c.) route with 10 μl of the bacterial inoculum (10^6^ cfu/mouse corresponding to a lethal dose killing 20% of animals, LD_20_). Mice were monitored for clinical signs according to a previously described coma scale [21] and euthanised if/when they reached a score of 2. Briefly, coma scale was as follows: 1 = coma, 2 = does not stand upright, 3 = stands upright within 30 sec, 4 = stands upright within 5 sec, minimal ambulatory activities, 5 = normal.

### Treatment with BB-94

Mice were treated i.p. with batimastat (BB-94; 50 mg/kg) 1 h before and 24 h post-infection. BB-94 (Merck Chemicals Ltd., Nottingham, United Kingdom) was suspended at 50 mg/ml in dimethylsulfoxide (DMSO) and stored frozen at -20°C. Prior to use, it was diluted 20-fold in phosphate buffered saline (PBS), and 500 μl were injected into animals. Control mice were injected with 500 μl of 5% DMSO in PBS. Animals were sacrificed 48 h after i.c. challenge.

### Experimental design and sample collection

Three independent experiments were performed with a total number of 41 mice (control, n = 20; treated, n = 21). Upon sacrifice, mice were deeply anaesthetised by i.p. injection with Zoletil 20 (30 mg/kg; Virbac Srl) and Xilor (8 mg/kg; Bio 98 Srl) and perfused transcardially with ice-cold PBS. Brains were removed and dissected into the two hemispheres and cerebellum. One hemisphere was fixed in 4% paraformaldehyde (PFA) in PBS (w/v) for histological analysis, while the other one was frozen in dry ice for assessment of intracerebral bleeding and BBB breakdown. Cerebella were frozen in dry ice for MMP and cytokine analysis. Samples were not collected from animals unsuccessfully perfused, found dead or humanely sacrificed before 48 h. Thus, analyses were conducted on a total of 13 control and 20 BB-94-treated mice.

### Brain histomorphometry

Cortical necrosis and apoptosis in the dentate gyrus of hippocampus were assessed as previously described [21]. Hemispheres were prepared for cryopreservation by incubation in 18% sucrose in PBS (w/v) at 4°C overnight. Forty-five μm-thick coronal sections obtained by random uniform sampling were stained with cresyl violet for Nissl substance. Histological features of apoptosis (condensed and/or fragmented dark nuclei, apoptotic bodies) were counted in 4 different slices spanning the hippocampus. Three visual fields in the two blades of the dentate gyrus were inspected. The number of apoptotic cells/visual field (apoptotic score) was determined, and a mean apoptotic score was calculated for each animal. Cortical damage was defined as areas of decreased neuronal density or frank cortical necrosis and evaluated on digitised brain section images using the ImageJ analysis software (U.S. National Institutes of Health, Bethesda, Maryland, USA) [31]. The volume of cortical damage was assessed in at least 16 brain sections/mouse and expressed as a percentage of the total cortical volume according to the Cavalieri principle as already described [32].

### Quantification of cyto-/chemokines and MMP-9

Cyto-/chemokines and MMP-9 in cerebella homogenates were quantified by using microsphere-based multiplex assays (Milliplex MAP mouse cytokine/chemokine multiplex assay and Milliplex MAP mouse CVD panel 1 multiplex assay, Merck Millipore, Billerica, MA, USA). Cerebella were homogenised with a glass Dounce homogeniser in 1:7 (w/v) extraction buffer containing 0.1% Triton X-100 and a protease inhibitor cocktail (Roche Diagnostics, Rotkreuz, Switzerland) in PBS. One hundred μg (for cyto-/chemokines) or 1 μg (for MMP-9) of homogenates were tested in duplicate. A minimum of 50 beads per analyte was measured using a Bio-Plex 200 station (Bio-Rad Laboratories, Hercules, CA, USA). Calibration curves from recombinant standards were calculated with the Bio-Plex Manager software version 4.1.1 using a five-parameter logistic curve fitting.

### Gelatin-sepharose affinity binding

Homogenised cerebella were centrifuged at 16000 x *g* for 10 min at 4°C, and the protein content was determined by the BCA protein assay (Pierce, Thermo Fischer Scientific, Reinach, Switzerland). Gelatinases were enriched by incubating 100 μg of homogenates with 20 μl of Gelatin Sepharose 4B (GE Healthcare GmbH, Glattbrugg, Switzerland) on an orbital shaker overnight at 4°C. Sepharose beads were washed (50 mM Tris-HCl pH 7.6, 150 mM NaCl, 5 mM CaCl_2_, 0.02% NaN_3_, 0.05% Brij 25, 1% Triton X-100) and then incubated with 2X zymography sample buffer (30 mM Tris HCl pH 6.8, 20% glycerol, 1% sodium dodecyl sulphate (SDS), 0.02% bromophenol blue) for 5 min at room temperature to elute bound proteins. After centrifugation, supernatants were collected and tested by gelatin gel zymography.

### Gelatin zymography

Gelatin zymography was performed as described [21]. Samples were subjected to electrophoresis under non-reducing conditions in polyacrylamide gels containing 1% (v/v) type A gelatin from porcine skin (Sigma-Aldrich). After electrophoresis, SDS was removed from the gels, and the MMP catalytic sites were activated by overnight incubation at 37°C in zymography buffer (10 mM CaCl_2_, 50 mM Tris, 50 mM NaCl, pH 7.65). Gels were stained with 0.1% Coomassie Brilliant Blue R250 (Sigma-Aldrich), 30% methanol and 10% acetic acid. Gelatinolytic activity was determined by densitometric quantification of the substrate lysis zones around 92 (pro-MMP-9) and 72 (pro-MMP-2) kDa using the ImageJ analysis software [31]. Purified human neutrophil MMP-9 and MMP-2 (Calbiochem, Darmstadt, Germany) were used as standards for normalisation and quantification of MMP-9 and MMP-2 as a function of the lysis zone. A strong correlation was calculated between MMP-9 quantification obtained by gel zymography and by the Milliplex assay (Pearson’s test, ρ = 0.83, *p* < 0.0001).

### Analysis of cerebral bleeding

Cerebral haemorrhages were assessed as previously described [33]. Briefly, brain hemispheres were cut in a frontal plane into 30 μm-thick sections, and serial sections were photographed with a digital camera at 0.3 mm-intervals. For each animal, 5 comparable brain sections were analysed. The bleeding spots were counted, and the relative areas of bleeding were measured by using the UTHSCSA Image Tool (Texas, USA). Cumulative bleeding areas were divided by the whole slice area and computed into a total bleeding area/whole slice area x 1000.

### Assessment of BBB integrity

The integrity of BBB was estimated by quantifying mouse serum albumin in brain homogenates by enzyme-linked immunosorbent assay (ELISA) as previously reported [34]. In brief, brain slices were dissolved in lysis buffer containing 0.1% NaN3 and homogenised in an ultrasound bath for 20 sec. After centrifugation, supernatants were collected and subjected to the Roti®-Nanoquant assay (Carl Roth GmbH + Co.KG, Karlsruhe, Germany) to quantify the total protein content. For each sample, 0.5 μg of total protein were subjected to albumin ELISA (Bethyl/Biomol GmbH, Hamburg, Germany) according to the manufacturer’s instructions.

### Statistical analyses

Results are presented as mean values ± standard deviation. Mouse survival was estimated by the Kaplan-Meier survival analysis, and differences were compared using the log-rank test (*p* < 0.05). Differences in MMP-9 gelatinolytic activity, apoptosis, BBB integrity and cerebral bleeding between mice treated with BB-94 and controls were evaluated using the Mann-Whitney U test (*p* < 0.05). Correlation was measured by the Pearson’s analysis (*p* < 0.05).

## Results

**Figure 1:**
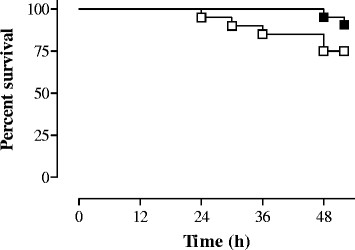
**Effect of treatment with BB-94 on survival of mice with MM.** BALB/c mice were infected i.c. with 10^6^ cfu/mouse of strain 93/4286 (group C) of *N. meningitidis,* injected i.p. with either BB-94 (n = 21) or vehicle (n = 20), and sacrificed 48 h later. Treatment with BB-94 (50 mg/kg) was carried out 1 h before and 24 h post-infection. Data are represented as a Kaplan-Meier survival curve.

The impact of treatment with BB-94 on MM outcome in infected mice was evaluated by assessing animal survival, brain damage, and levels of MMPs and cyto-/chemokines 48 h after meningococcal challenge. Upon sacrifice, the clinical scores of surviving mice were 3 (2-4) [median (range)] irrespective of the group (*p* > 0.05). Kaplan-Meier analysis of survival (at 48 h) showed that animals treated with BB-94 had increased survival (95.2%) in comparison with controls (75%), and differences were almost statistically significant (*p* = 0.064), (Figure 1). The concentrations of interleukin (IL)-1β, IL-10, monocyte chemoattractant protein 1 (MCP-1) and macrophage inflammatory protein 1α (MIP-1α) were slightly reduced in cerebella of treated animals, without reaching statistical significance (data not shown).

### BB-94 reduces MMP-9 levels in the brain of mice with MM

**Figure 2:**
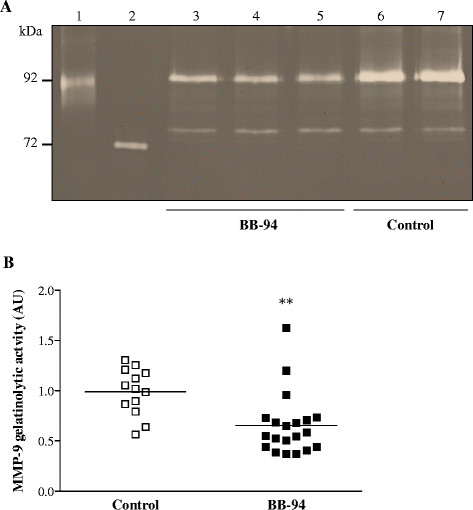
**Zymography on cerebella of control and BB-94-treated mice infected with meningococci.** Mice were infected and treated as described in Figure 1. Animals were perfused with cold PBS, and cerebella were harvested and homogenised. As samples were not collected from dead or unsuccessfully perfused animals, a total of 20 treated mice and 13 control were analysed. Gelatinases in cerebella were enriched by incubation with gelatin-sepharose and subjected to gelatin zymography. **A**. Densitometry of gelatinolytic activity around 92 kDa and 72 kDa. Lane 1: human MMP-9; lane 2: human MMP-2; lanes 3-5: BB-94-treated mice; lanes 6-7: controls. **B**. Quantification of MMP-9 gelatinolytic activity. Results are combined from 3 independent experiments. AU, arbitrary units. **p < 0.01.

To evaluate whether BB-94 diminished the levels of MMPs in the brain, gelatin zymography was performed on cerebella of treated and control animals after MM induction. Densitometric analysis showed no relevant changes in MMP-2 levels between samples from the two mouse groups. In contrast, the amount of MMP-9 was diminished in cerebella of mice that had received BB-94 compared to those injected with vehicle (Figure 2A). Quantitative assessment of MMP-9 based on densitometric analysis of MMP-9 substrate lysis zones evidenced a significant reduction (*p* = 0.0011) between treated (0.6523 ± 0.3079) and control (0.9893 ± 0.2310) mice (Figure 2B).

### Treatment with BB-94 ameliorates intracranial complications in infected mice

**Figure 3:**
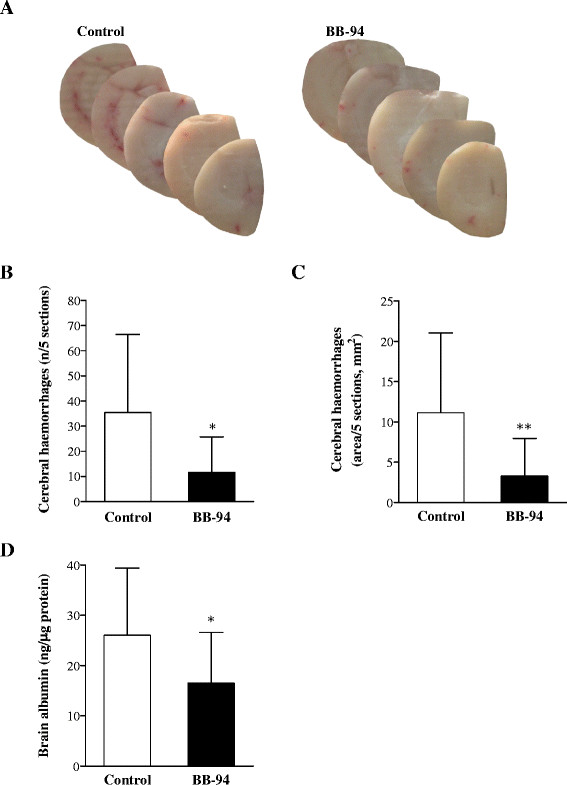
**Brain damage in control and BB-94-treated mice after meningococcal challenge.** Mice were infected and treated as described in Figure 1. Brains from perfused animals were collected. **A-C**. Brain hemispheres were cut in 30 μm cryosections and photographed to determine the number of haemorrhagic spots and the areas of bleeding. Macroscopical assessment of cerebral haemorrhages in animals injected with BB-94 or vehicle **(A)**. Enumeration of bleeding spots **(B)** and measurement of haemorrhagic areas **(C)** were carried out on 5 comparable brain sections/mouse. **D**. Following bleeding assessment, brain hemispheres were homogenised, and supernatants were subjected to mouse albumin ELISA to quantify serum albumin in the brain as a marker of BBB breakdown. Data are displayed as ng of albumin per μg of total protein content of the brain. Data are from 3 independent experiments. *p < 0.05; **p < 0.01.

Preliminary observations upon MM model development indicated that mice infected with *N. meningitidis* presented multifocal cortical haemorrhages visible to the naked eye compared to naïve animals (Ricci, unpublished data). Therefore, the number and size of cerebral bleedings in BB-94-treated mice were compared to those from control subjects. Macroscopical examination of brain samples showed reduced bleeding in animals treated with BB-94 in comparison with controls (Figure 3A). Quantitative analysis revealed a 3-fold reduction both in the number of haemorrhagic spots (Figure 3B; *p* = 0.0122) and the area of bleeding (Figure 3C; *p* = 0.0061) in treated mice. However, cortical haemorrhages did not translate into large regions of reduced neuronal density as detectable by Nissl staining (data not shown).

BB-94 was also beneficial for the integrity of the BBB, as proven by reduced diffusion of serum albumin in the brain of treated mice in respect to those injected with vehicle. Differences between the concentration of albumin in BB-94-treated (16,47 ± 10,11 ng/μg total protein) and control (26,00 ± 13,37 ng/μg) animals were statistically significant (*p* = 0.037; Figure 3D).

Evaluation of apoptosis in the dentate gyrus of hippocampus showed a trend in the reduction of the apoptosis score in the treatment group (0,1438 ± 0,1396) compared to the control one (0,1898 ± 0,1217; *p* > 0.05).

### MMP-9 contributes to brain damage associated with MM

**Figure 4:**
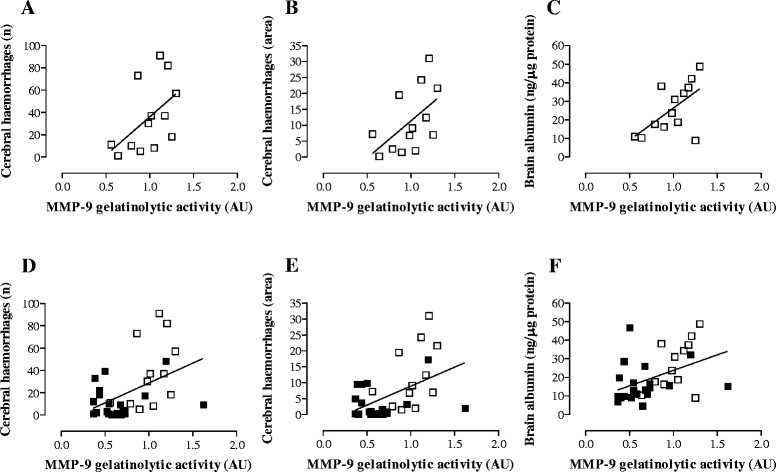
**Correlation between MMP-9 levels and intracranial complications in mice with MM.** Brain samples from control and treated animals were subjected to different assays as described in Figures 2 and 3. **A-C**. Control mice (open squares). **D-F**. Pooled data of control (open squares) and BB-94-treated animals (closed squares). The amount of MMP-9 assessed by zymography was associated with the number of bleeding spots **(A, D)**, the size of haemorrhages **(B, E)**, and the disruption of BBB **(C, F)**. Correlation analysis was performed by the Pearson’s test (*p* < 0.05).

To evaluate whether levels of MMP-9 are associated with intracranial complications resulting from MM, Pearson correlation analysis was performed on data from both control and BB-94-treated mice (Figure 4 and Additional file 1: Table S1). In infected control animals, the amounts of MMP-9 significantly correlated with the extent of BBB disruption (ρ = 0.59, *p* = 0.031; Figure 4C). Correlation between MMP-9 levels and the number of haemorrhagic spots (ρ = 0.51, *p* = 0.079) or the size of bleeding (ρ = 0.54, *p* = 0.054) also showed a tendency towards statistical significance (Figure 4A-B). In contrast, no significant correlation was observed between amounts of MMP-9 and intracranial complications in treated mice (Additional file 1: Table S1). Finally, when data from both control and BB-94-treated animals were pooled, MMP-9 levels presented a robust correlation with the number (ρ = 0.46, *p* = 0.0067; Figure 4D) and area (ρ = 0.47, *p* = 0.0049; Figure 4E) of cerebral bleeding, and with the breakdown of BBB (ρ = 0.44, *p* = 0.0104; Figure 4F).

## Discussion

Successful treatment of BM not only requires antibiotics to eradicate the pathogen but also adjuvant therapeutic molecules targeting the host immune and inflammatory responses. Potential targets of adjuvant therapy are cyto- and chemokines, leukocytes, coagulation cascade molecules, oxidants, caspases, and degrading enzymes [35],[36]. To this purpose, several therapeutic approaches have been attempted in animal models of meningitis caused by *Streptococcus pneumoniae* and *Streptococcus agalactiae*, including the use of nonbacteriolytic antibiotics, antioxidants, antagonists of excitatory amino acids, and also inhibitors of transcription factors, MMPs, caspases, or complement factors [17]-[21],[33],[37]-[41]. In contrast, data on adjuvant therapy in MM animal models are scant partially due to limitations of experimental systems mimicking the human disease.

In this work, we have fine-tuned a recently described MM model [30] to study brain damage associated with MM in the mouse. The model is based on i.c. infection of adult mice with a sub-lethal dose (~LD_20_) of live meningococci in order to limit a rapid fatal outcome and permit the development of brain damage. Model readouts included animal survival and clinical signs, cyto-/chemokines profiling, MMP levels, apoptosis, BBB breakdown, and intracerebral haemorrhages. Compared to some experimental model of PM [14],[15], animals were not treated with antibiotics, which may influence the course and profile of the inflammatory response. The availability of a simple and reliable MM mouse model allowed, for the first time, to provide evidence that MMP-9 is involved in the pathogenesis of MM-induced brain damage and MMP-9 inhibition significantly improves intracranial complications.

Common histopathologic features of PM include brain oedema, cortical haemorrhages, focal necrotic lesions in cortical/subcortical structures and neuronal apoptosis in the hippocampus [6],[7]. PM animal models have largely proven the role of MMPs in several mechanisms of brain damage, including PMN extravasation, neuronal damage and BBB opening [14]-[16]. Concerning MM models, challenge of rats with heat-killed meningococci increased the levels of MMP-9 mRNA expression and proteolytic activity [22] and contributed to BBB disruption [11]. A recent study demonstrated that infection of human brain microvascular endothelial cells with *N. meningitidis* induced both the detachment of brain endothelial cells from the matrix and the cleavage of the tight junction protein occludin; interestingly, both processes were mediated by MMP-8, thereby proposing a molecular mechanism behind BBB breakdown during MM [42]. However, these studies were carried out either *in vivo* with inactivated bacteria [11],[22] or *in vitro* [42]. Our work, instead, employed live meningococci in mice and provided *in vivo* evidence that MMP-9 levels significantly correlated with BBB breakdown and cerebral bleeding.

The blood vessel is a major target of both pathogen- and host-mediated injury in MD. During haematogenous spread, *N. meningitidis* interacts with the endothelial cells of blood vessels throughout the body, including brain (in MM) and skin (in sepsis). Recently, the skin lesions typical of *purpura fulminans* were reproduced in an experimental model of meningococcemia [43], demonstrating how ‘vascular colonisation’ by meningococci initiates the thrombotic process [44] that ultimately leads to vasculitis and vascular rupture. A similar mechanism may occur in the blood vessels of the brain during experimental MM, resulting in cerebral bleeding. On the host side, MMPs also contribute to the dysfunction of brain vasculature by disrupting endothelial cell junctions [42] and promoting BBB opening [11]. MMPs participate in cerebral haemorrhage of diverse origins [45], and blocking MMPs is an effective strategy to control brain (vasogenic) oedema and bleeding size in animal models of intracerebral haemorrhage [46]-[49]. In our case, the correlation between MMP-9 levels and bleeding is consistent with a key role of MMPs in brain haemorrhage, suggesting that both meningococci and MMPs may have contributed to intracerebral bleeding in the model.

Inhibitors of MMPs have shown beneficial effects in experimental studies of neuroinflammatory and neurodegenerative conditions, including multiple sclerosis, ischaemic and haemorragic stroke, vascular dementia and meningitis [9],[10]. In an infant rat PM model, several molecules with different inhibitory profiles (MMPs or TNF-α converting enzyme (TACE)) were able to reduce animal mortality [19],[21], cortical necrosis [15],[17]-[19],[21], hippocampal apoptosis [17],[21], BBB disruption [19], cyto-/chemokine release [21], and also post-infection sequelae [17]-[19]. Batimastat (BB-94) is a hydroxamate peptidomimetic broad-spectrum MMP inhibitor, but inactive against TACE. Screening of a wide range of different MMP inhibitors in a lipopolysaccharide (LPS)-based rodent model showed that BB-94 was the most effective compound at reducing BBB breakdown in both rats and mice [50]. BB-94 was also useful to diminish BBB disruption and intracranial pressure in rats infected with heat-killed *N. meningitidis* [11]. Moreover, BB-94 significantly reduced the incidence of haemorrhage associated with administration of thrombolytic drugs in rabbits suffering from stroke, demonstrating the added value of BB-94 in controlling experimental brain bleeding [46].

In this study, treatment with BB-94 increased mouse survival and alleviated the severity of intracranial complications. Kaplan-Meier survival curves showed a tendency towards a significant difference between treated and control animals. The lack of statistical significance may be due to the relatively small sample size of animal groups affecting statistical power. In contrast, brain damage was significantly reduced in BB-94-treated subjects compared to controls. To emphasise this result, it is worth noticing that mice (of which 87.5% were controls) that died before the experimental endpoint at 48 h were not assessed for brain injury, thereby potentially excluding the most acute cases from the analyses. BB-94 may have operated via different mechanisms, including: (i) a direct effect on the brain microvasculature by limiting vascular rupture and BBB breakdown, or (ii) an indirect action on the systemic inflammatory response by reducing MMP-dependent activation of pro-inflammatory mediators. However, the fact that levels of brain cyto- and chemokines were not significantly reduced in treated mice argues for a direct vasculoprotective effect by BB-94. In BB-94 treated mice, MMP-9 levels did not significantly correlate with intracerebral complications, suggesting that other factors than MMP-9 may be responsible for the residual brain damage detectable in treated animals.

Drugs that block MMPs are considered a double-edged sword in clinical practice. On one hand, they represent a therapeutic asset in neuroinflammation, while on the other, they may wedge crucial recovery functions, such as neurovascular remodelling and neurogenesis [10]. Nevertheless, MM is an acute condition for which a short-term adjuvant therapy with MMP inhibitors may be envisioned to protect the neurovascular unit.

## Conclusions

This study describes the development of a novel murine model of MM-induced brain damage that allows the assessment of different clinical, histopathological and immunological parameters. The model may be valuable in the MM/MD field where experimental systems mimicking the human disease are lacking. The use of such *in vivo* model enabled us to demonstrate that: (i) MMP-9 contributes to neuronal injury in MM, and (ii) MMP-9 inhibition improves disease outcome, indicating that MMP-9 may represent an important target for adjuvant therapy in MM. The present data are consistent with the literature highlighting the role of MMPs in PM, thus suggesting MMPs as universal mediators of brain damage in acute BM irrespective of the causative agent. Future studies will investigate the efficacy of MMP inhibitors as adjuvant therapy in combination with antibiotics in experimental MM.

## Additional file

## Electronic supplementary material

Additional file 1: Table S1: Describing the statistical analysis related to correlation between zymography and brain damage in mice with MM. (DOCX 27 KB)

Below are the links to the authors’ original submitted files for images.

Authors’ original file for figure 1

Authors’ original file for figure 2

Authors’ original file for figure 3

Authors’ original file for figure 4

## Abbreviations

BM: Bacterial meningitis
MD: Meningococcal disease
MM: Meningococcal meningitis
PM: Pneumococcal meningitis
MMP: Matrix metalloproteinase
TNF-α: Tumor necrosis factor α
IL: Interleukin
MCP-1: Monocyte chemoattractant protein 1
MIP-1α: Macrophage inflammatory protein 1α
TACE: TNF-α converting enzyme
PMN: Polymorphonuclear cell
BBB: Blood-brain barrier
CSF: Cerebrospinal fluid
LPS: Lipopolysaccharide
i.c.: Intracisternally
i.p.: Intraperitoneally
LD_20_: Lethal dose killing 20% of animals
DMSO: Dimethylsulfoxide
PBS: Phosphate buffered saline
PFA: Paraformaldehyde
SDS: Sodium dodecyl sulphate
ELISA: Enzyme-linked immunosorbent assay
