# Supplementary material for: Inhibition of matrix metalloproteinases attenuates brain damage in experimental meningococcal meningitis
Source: BMC Infect Dis. 2014 Dec 31;14:726. doi: 10.1186/s12879-014-0726-6 (PMC4300156; doi:10.1186/s12879-014-0726-6)
Supplement: Supplementary file 1 — Additional file 1: Table S1: Describing the statistical analysis related to correlation between zymography and brain damage in mice with MM. (DOCX 27 KB) [file 12879_2014_726_MOESM1_ESM.docx]

**Additional file 1: Table S1. Correlation analysis between zymography and brain damage in mice infected with *N. meningitidis*.**

| **Group (n) ^a^** | **MMP-9/no. of bleeding** | | **MMP-9/bleeding area** | | | | **MMP-9/BBB disruption** | |
| --- | --- | --- | --- | --- | --- | --- | --- | --- |
|  | **Pearson ρ ^b^** | ***p* value** |  | **Pearson ρ** | ***p* value** |  | **Pearson ρ** | ***p* value** |
| **DMSO (13)** | 0.51 | 0.079 |  | 0.54 | 0.054 |  | 0.59 | 0.031**^*^** |
| **BB-94 (20)** | 0.14 | 0.54 |  | 0.14 | 0.54 |  | 0.14 | 0.55 |
| **Pooled (33)** | 0.46 | 0.0067**^**^** |  | 0.47 | 0.0049**^**^** |  | 0.44 | 0.0104**^*^** |

^a^ Mice were infected i.c. with *N. meningitidis* and injected i.p. with BB-94 (n=20) or vehicle (DMSO, n=13). Animals were sacrificed 48 h post-infection, brains were collected and subjected to different assays to assess MMP-9 levels, intracerebral haemorrhage, and BBB disruption. For each mouse, levels of MMP-9 were correlated with the number of bleeding spots, bleeding areas, and the extent of BBB disruption.

^b^ Correlation was measured by the Pearson analysis (ρ, Pearson’s correlation coefficient). *P* values <0.05 were considered as significant (**^*^**, *p*<0.05; **^**^**, *p*<0.01). Analyses were performed on the groups of control (n=13) and BB-94-treated (n=20) mice separately, and also on pooled data (n=33).
