# Supplementary figures and images for: Inhibition of matrix metalloproteinases attenuates brain damage in experimental meningococcal meningitis
Source: BMC Infect Dis. 2014 Dec 31;14:726. doi: 10.1186/s12879-014-0726-6 (PMC4300156; doi:10.1186/s12879-014-0726-6)

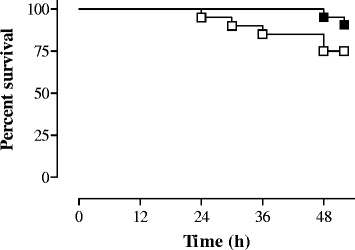

Supplement: Supplementary file 2 — Authors’ original file for figure 1 [file 12879_2014_726_MOESM2_ESM.gif]

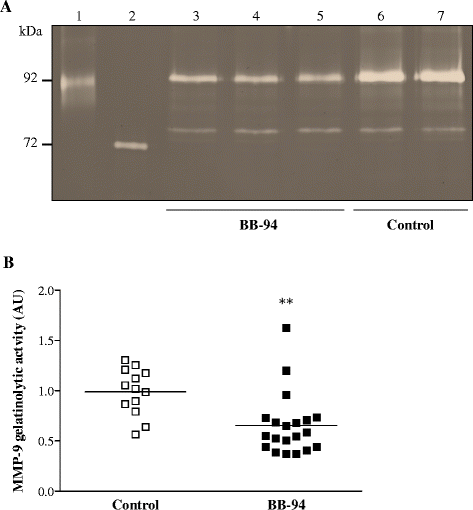

Supplement: Supplementary file 3 — Authors’ original file for figure 2 [file 12879_2014_726_MOESM3_ESM.gif]

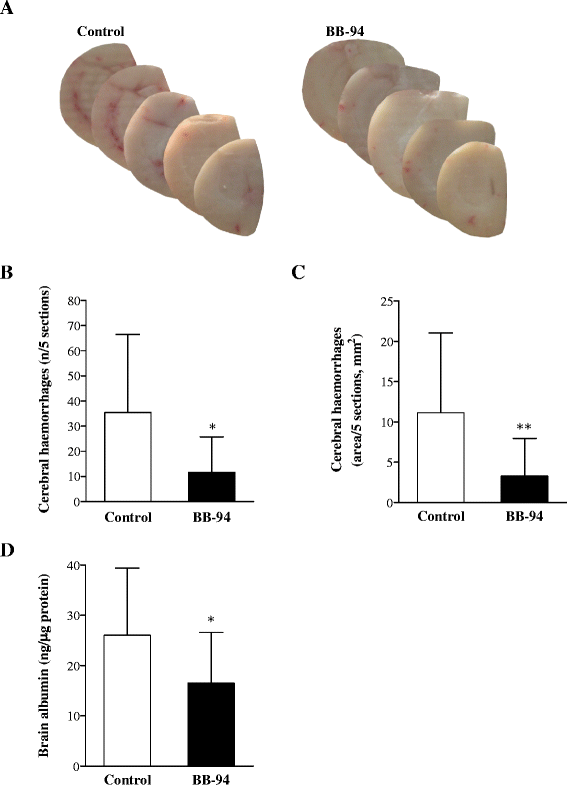

Supplement: Supplementary file 4 — Authors’ original file for figure 3 [file 12879_2014_726_MOESM4_ESM.gif]

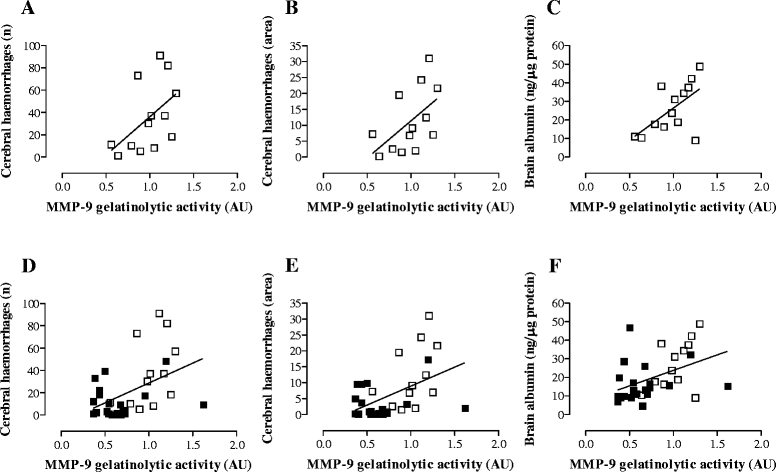

Supplement: Supplementary file 5 — Authors’ original file for figure 4 [file 12879_2014_726_MOESM5_ESM.gif]
